# A three-pocket model for substrate coordination and selectivity by the nucleotide sugar transporters SLC35A1 and SLC35A2

**DOI:** 10.1016/j.jbc.2021.101069

**Published:** 2021-08-10

**Authors:** Danyang Li, Somshuvra Mukhopadhyay

**Affiliations:** Division of Pharmacology & Toxicology, College of Pharmacy, Institute for Cellular & Molecular Biology, and Institute for Neuroscience, University of Texas at Austin, Austin, Texas, USA

**Keywords:** nucleotide sugar transporters, substrate specificity, structural determinants, rescue assays, mutagenesis, three-pocket model, FACS, fluorescence-activated cell sorting, NST, nucleotide sugar transporter, PNA, peanut agglutinin, WT, wild-type

## Abstract

The CMP-sialic acid transporter SLC35A1 and UDP-galactose transporter SLC35A2 are two well-characterized nucleotide sugar transporters with distinctive substrate specificities. Mutations in either induce congenital disorders of glycosylation. Despite the biomedical relevance, mechanisms of substrate specificity are unclear. To address this critical issue, we utilized a structure-guided mutagenesis strategy and assayed a series of SLC35A2 and SLC35A1 mutants using a rescue approach. Our results suggest that three pockets in the central cavity of each transporter provide substrate specificity. The pockets comprise (1) nucleobase (residues E52, K55, and Y214 of SLC35A1; E75, K78, N235, and G239 of SLC35A2); (2) middle (residues Q101, N102, and T260 of SLC35A1; Q125, N126, Q129, Y130, and Q278 of SLC35A2); and (3) sugar (residues K124, T128, S188, and K272 of SLC35A1; K148, T152, S213, and K297 of SLC35A2) pockets. Within these pockets, two components appear to be especially critical for substrate specificity. Y214 (for SLC35A1) and G239 (for SLC35A2) in the nucleobase pocket appear to discriminate cytosine from uracil. Furthermore, Q129 and Q278 of SLC35A2 in the middle pocket appear to interact specifically with the β-phosphate of UDP while the corresponding A105 and A253 residues in SLC35A1 do not interact with CMP, which lacks a β-phosphate. Overall, our findings contribute to a molecular understanding of substrate specificity and coordination in SLC35A1 and SLC35A2 and have important implications for the understanding and treatment of diseases associated with mutations or dysregulations of these two transporters.

Nucleotide sugar transporters (NSTs) transport nucleotide sugars from the cytosol into the lumen of the endoplasmic reticulum or the Golgi apparatus, where the nucleotide sugars serve as substrates for protein glycosylation and glycosphingolipid synthesis (1). NSTs belong to the SLC35 family and are evolutionarily conserved (1, 2, 3). Currently, 31 SLC35 members have been identified in the human genome. SLC35 members are divided into seven subfamilies, from SLC35A to SLC35G (3). They transport a wide range of nucleotide sugars, including CMP-sialic acid, UDP-glucose, UDP-galactose, etc (1, 3). Some NSTs can transport more than one substrate and some substrates can be recognized by more than one NST. Considering the structural similarities shared among nucleotide sugars, NSTs have to possess explicit mechanisms that allow them to discriminate between different substrates.

Two of the best-characterized NSTs are CMP-sialic acid transporter SLC35A1 and UDP-galactose transporter SLC35A2 (3, 4). The human protein sequences of the two transporters are ∼46% identical. Loss of SLC35A1 activity leads to the lack of sialylation on N-glycosylated proteins. Deficiency in SLC35A2 inhibits galactosylation on N-glycosylated proteins and eliminates most of the sialyation sites on N-glycans. Pathogenic mutations in SLC35A1 or SLC35A2 respectively cause congenital disorders of glycosylation type IIf or type IIm. The central nervous system is the most affected, which may be because of the role of N-glycans in modulating neural transmission and the excitability of neural circuits (5, 6). In addition to congenital disorders of glycosylation, NSTs are also implicated in other diseases and may serve as good therapeutic targets. Some NSTs are host or virulence factors required for infections by parasites, viruses, or bacteria. As examples, the Golgi GDP-mannose transporter of the protozoan parasite *Leishmania* is required for its virulence (7); human host factor SLC35A1 is required for the entry of influenza A virus into cells (8); and human host factor SLC35A2 is required for the transport and toxicity of bacterial Shiga toxin 1 and 2 (STx1 and STx2; see below) (9, 10). NSTs may be inhibited by nucleoside analog antiviral chemotherapies and account for their adverse effects (11). Additionally, NSTs can also be targeted to reduce cancer cell hypersialylation, which promotes tumor survival and proliferation (12, 13). Information on the structural basis for substrate selectivity and coordination would be valuable to understand the pathobiology of diseases associated with NST function and activity, as well as contribute toward the development of specific NST modulators.

Most of our understanding of the substrate selectivity and coordination mechanisms of NSTs comes from a set of structure–function studies (14, 15, 16, 17, 18). NSTs are transmembrane proteins with ten transmembrane helices, with both N- and C-termini exposed to the cytosol (19). To date, the crystal structures of three NSTs, yeast GDP-mannose transporter Vrg4 (20, 21), maize CMP-sialic acid transporter (22), and murine SLC35A1 (23) have been reported. In maize CMP-sialic acid transporter, residues E42, K45, Y82, N86, K108, Y199, and K262 (corresponding to E52, K55, Y98, N102, K124, Y214, and K272 of mammalian SLC35A1) coordinate CMP and are required for optimal activity (22). Moreover, the crystal structure of murine SLC35A1 revealed that residues K55, Q101, N102, Y117, Y121, K124, S188, N210, Y214, T260, and K272 form polar interactions with CMP-sialic acid (23). Though crystal structures of the two CMP-sialic acid transporters provide insights into the mechanisms of CMP-sialic acid coordination by CMP-sialic acid transporters, no crystal structure is available for UDP-galactose transporters, and little is known about how the two most distantly related transporters differentiate between different substrates.

Our interest in studying NSTs emerged from the discovery that SLC35A2 is required for the trafficking and toxicity of bacterial STx1 and STx2, which cause lethal food borne disease and currently lack treatments (9, 10). UDP-galactose transported by SLC35A2 into the Golgi acts as a substrate for the production of globotriaosylceramide (Gb3), which is the cell surface receptor for the B-subunits of STx1/STx2 (STx1B/STx2B) (10). Previously, we used the requirement of SLC35A2 for the production of Gb3 and surface binding of STx1B/STx2B to develop a rescue assay and analyzed a series of structural and disease-associated mutations of SLC35A2 (10). Through this, we identified several amino acid residues that are critical for SLC35A2 activity, including K78, S213, and K297. Here, our goal was to leverage the rescue approach to gain insights into the mechanisms that confer substrate specificity to SLC35A2 and SLC35A1. The long-term perspective was that understanding the mechanism of substrate specificity would allow for the development of highly specific modulators that target SLC35A2 or SLC35A1 and that may be used for the treatment of Shiga toxicosis and other diseases associated with mutations or dysregulations of these two NSTs. This line of investigation was also expected to advance understanding of NST substrate specificity more generally.

## Results

### Identification of critical solvent-exposed residues required for SLC35A2 activity

**Figure 1:**
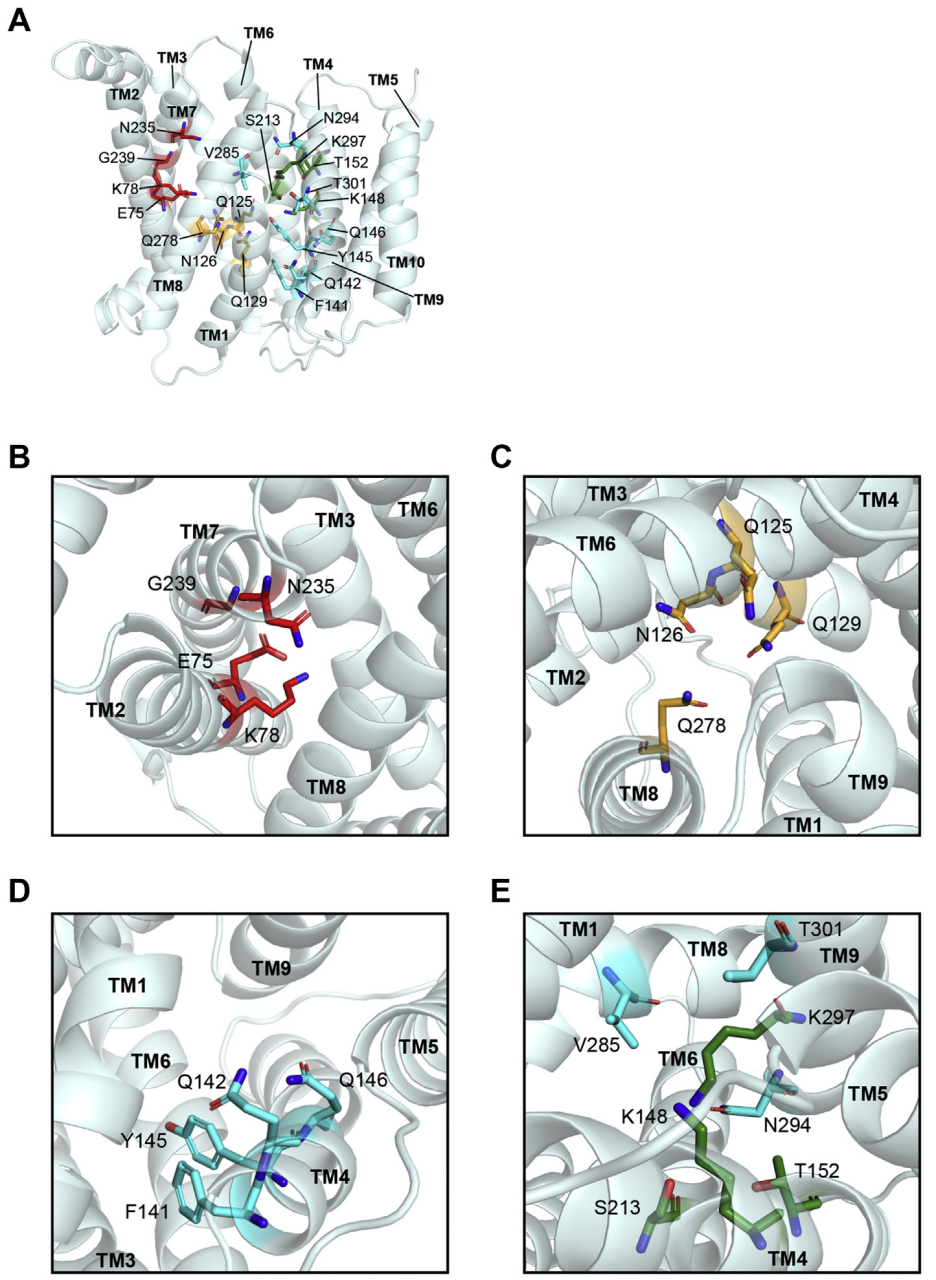
**Predicted structure of SLC35A2 with targeted critical residues.***A–E*, Cartoon depiction of the predicted structure of SLC35A2. Residues targeted for analyses are depicted as *colored sticks* (three residues that were analyzed in our previous study (10), K78, S213, and K297 are also presented). Color coding similar to Figure 8. Residues not involved in any of the three pockets (see below) are colored in *cyan*. TM, transmembrane helix.

We had previously used the yeast GDP-mannose transporter to obtain a predicted structure of SLC35A2 for functional analyses (10). In this study, we improved the prediction of the SLC35A2 structure (obtained using the PHYRE 2.0 algorithm) by utilizing the recently solved crystal structure of a more relevant NST, murine SLC35A1 (23, 24). We initiated our studies by focusing on 17 residues exposed to the central cavity, most of which are hydrophilic and/or charged (Fig. 1, *A*–*E*). All were substituted with alanine, except G239, which was replaced with tyrosine because the corresponding residue in SLC35A1 is tyrosine that may contribute to substrate specificity.

**Figure 2:**
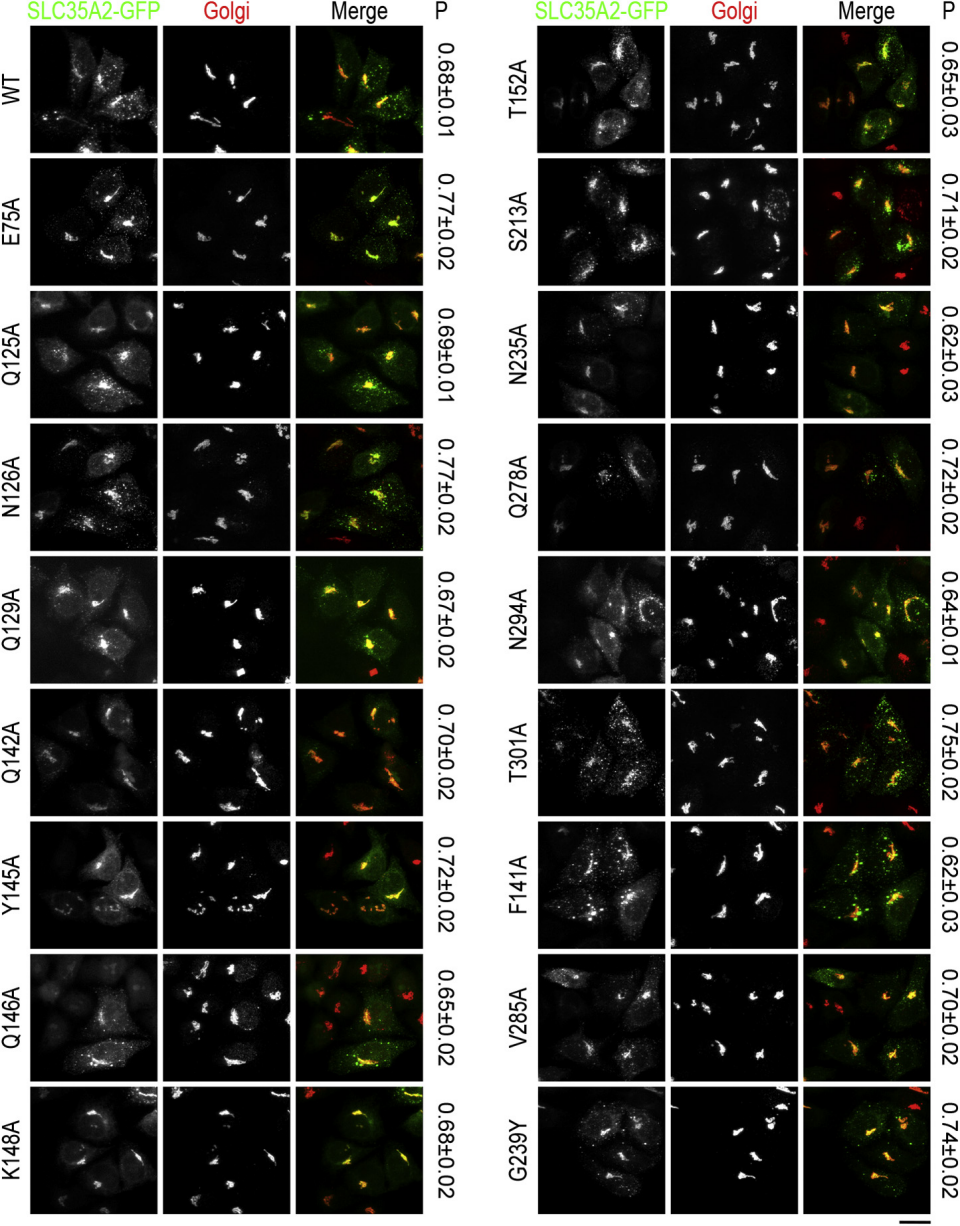
**Localization of central cavity-exposed SLC35A2 mutants in *ΔSLC35A2* cells.***ΔSLC35A2* cells were transfected with indicated SLC35A2-GFP constructs. Twenty-four hours after transfection, cultures were imaged to detect GFP and giantin (to demarcate the Golgi apparatus). “P” denotes values for the Pearson's coefficient for colocalization between two channels (mean ± SEM, n ≥ 28 cells per SLC35A2 construct). Scale bar, 20 μm.

Quantitative immunofluorescence using the Pearson's coefficient for colocalization revealed that all mutants generated were targeted to the Golgi apparatus, similar to the wild-type (WT) protein (Fig. 2; see Experimental procedures for details). As mutations that induce misfolding are expected to lead to the retention of the protein in the endoplasmic reticulum (10), the localization data suggests that the mutations introduced here did not have major impacts on folding.

**Figure 3:**
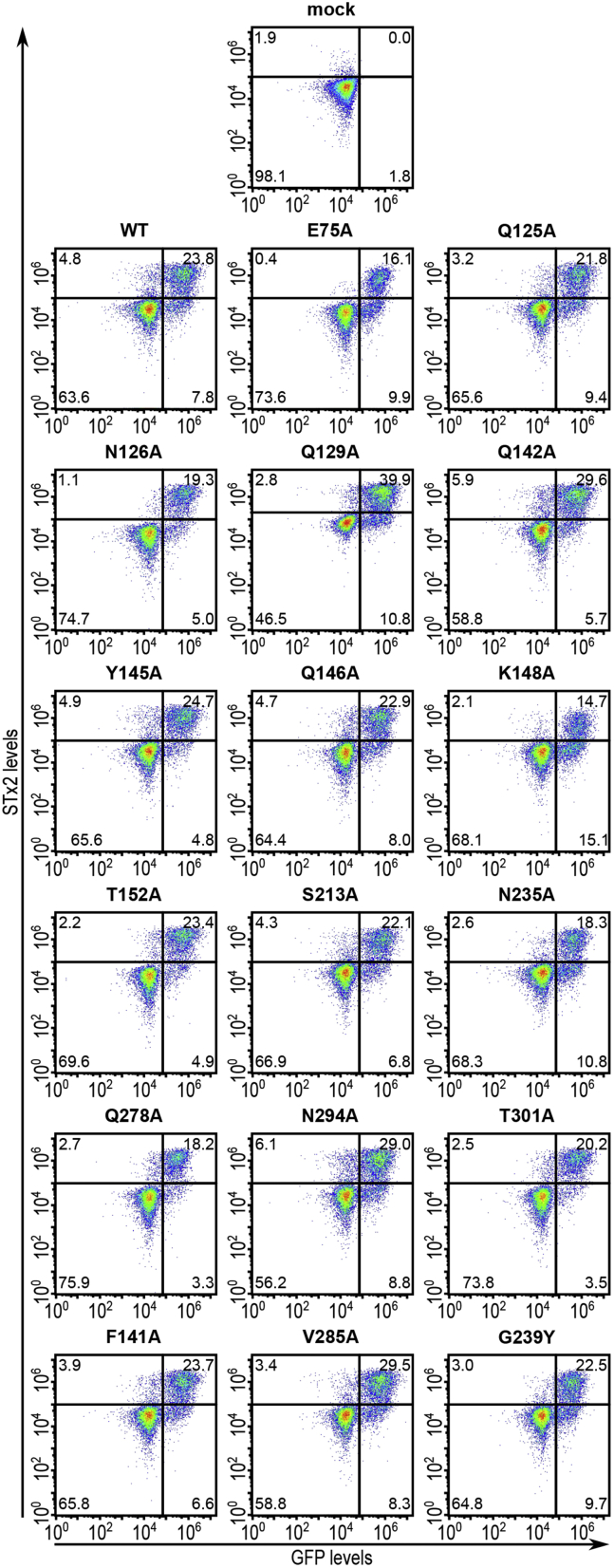
**Functional analyses of critical solvent-exposed SLC35A2 mutants.***ΔSLC35A2* cells were mock-transfected or transfected with indicated SLC35A2-GFP constructs. Twenty-four hours after transfection, cultures were processed to assay for STx2B binding by flow cytometry. Data from one replicate is depicted here and from multiple replicates quantified in Figure 4.

**Figure 4:**
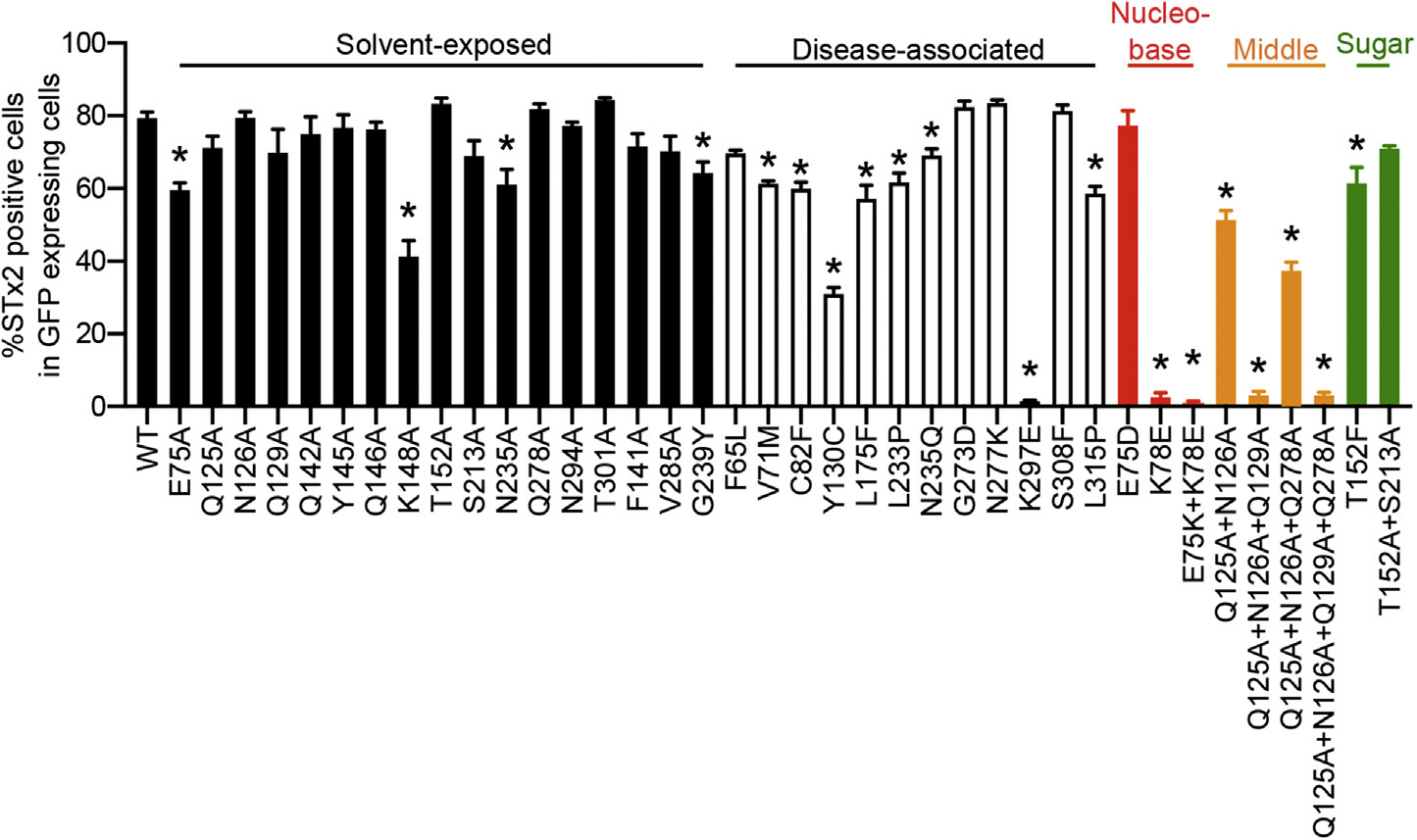
**Quantification of cell percentages positive for STx2B.** In GFP-expressing cells from Figures 3, 7, and 9*B*, cell percentages positive for STx2B binding were quantified (mean ± SE; n ≥ 3; ∗*p* < 0.05 for the comparison between WT SLC35A2-GFP and all other constructs by one-way ANOVA and Dunnett's *post hoc* test).

We assayed for the activity of the mutants by determining their capability to rescue STx2B binding in *ΔSLC35A2* HeLa cells, which we previously generated using CRISPR/Cas9 and have extensively described and validated (10). As expected, WT SLC35A2 expression rescued STx2B binding (Fig. 3). Similarly, Q125A, N126A, Q129A, Q142A, Y145A, Q146A, T152A, S213A, Q278A, N294A, T301A, F141A, and V285A fully restored STx2B binding (Figs. 3 and 4, note here that Figure 4 also shows quantification results for other SLC35A2 mutants assayed below) indicating that those residues are not required for SLC35A2 activity. However, E75A, N235A, G239Y, and K148A had reduced rescue activity (Figs. 3 and 4), indicating that these residues are necessary for activity. Interestingly, we previously reported that, unlike the S213A mutation, a phenylalanine substitution at S213 (S213F) strongly inhibited SLC35A2 activity (10). We further extend this observation in a subsequent part of the Results section by assaying for the function of the size of the S213 residue.

All SLC35A2 mutants in this study, including those described above, were C-terminally tagged with GFP. We previously reported that the GFP fusion does not interfere with SLC35A2 activity (10). We further validated this result here by observing that the ability of WT SLC35A2-GFP to rescue STx2B binding was comparable to N-terminally FLAG-tagged SLC35A2 (Fig. S1, *A* and *B*). Furthermore, in the rescue experiment, binding of STx2B in *ΔSLC35A2* cells expressing either epitope-tagged construct was comparable to mock-transfected WT cells (Fig. S1, *A* and *B*). Thus, WT GFP-tagged SLC35A2 fully rescues SLC35A2 activity.

### Effects of disease-associated mutations on SLC35A2 activity

**Figure 5:**
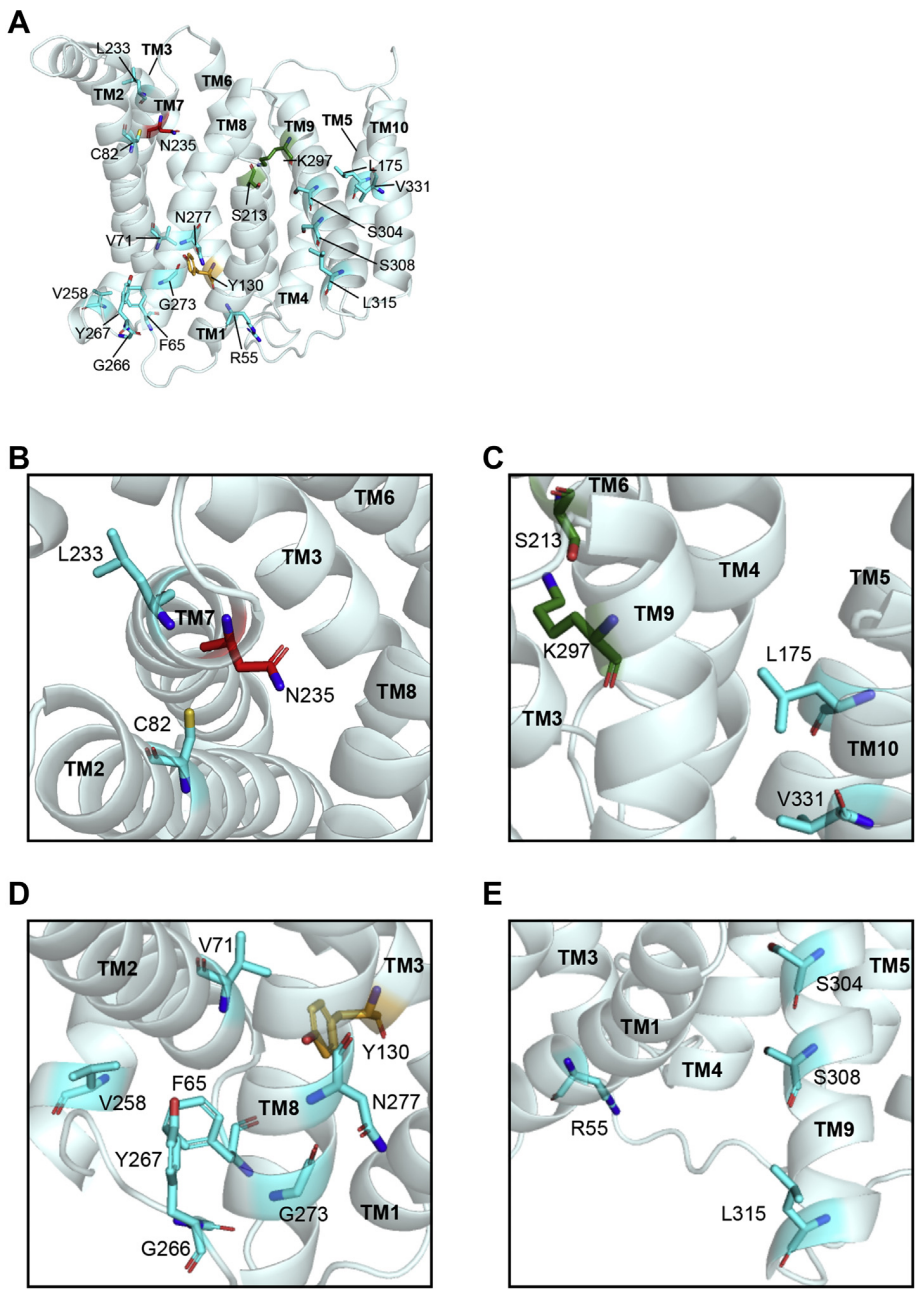
**Depiction of disease-associated residues on the predicted structure of SLC35A2.***A–E*, Disease-associated residues targeted for mutational analyses are depicted as *colored stick*s (note that eight residues from our previous study (10), R55, S213, V258, G266, Y267, K297, S304, and V331 are also presented). Color coding is similar to Figure 8, and residues not involved in any of the three pockets (see below) are colored in cyan. TM, transmembrane helix.

**Figure 6:**
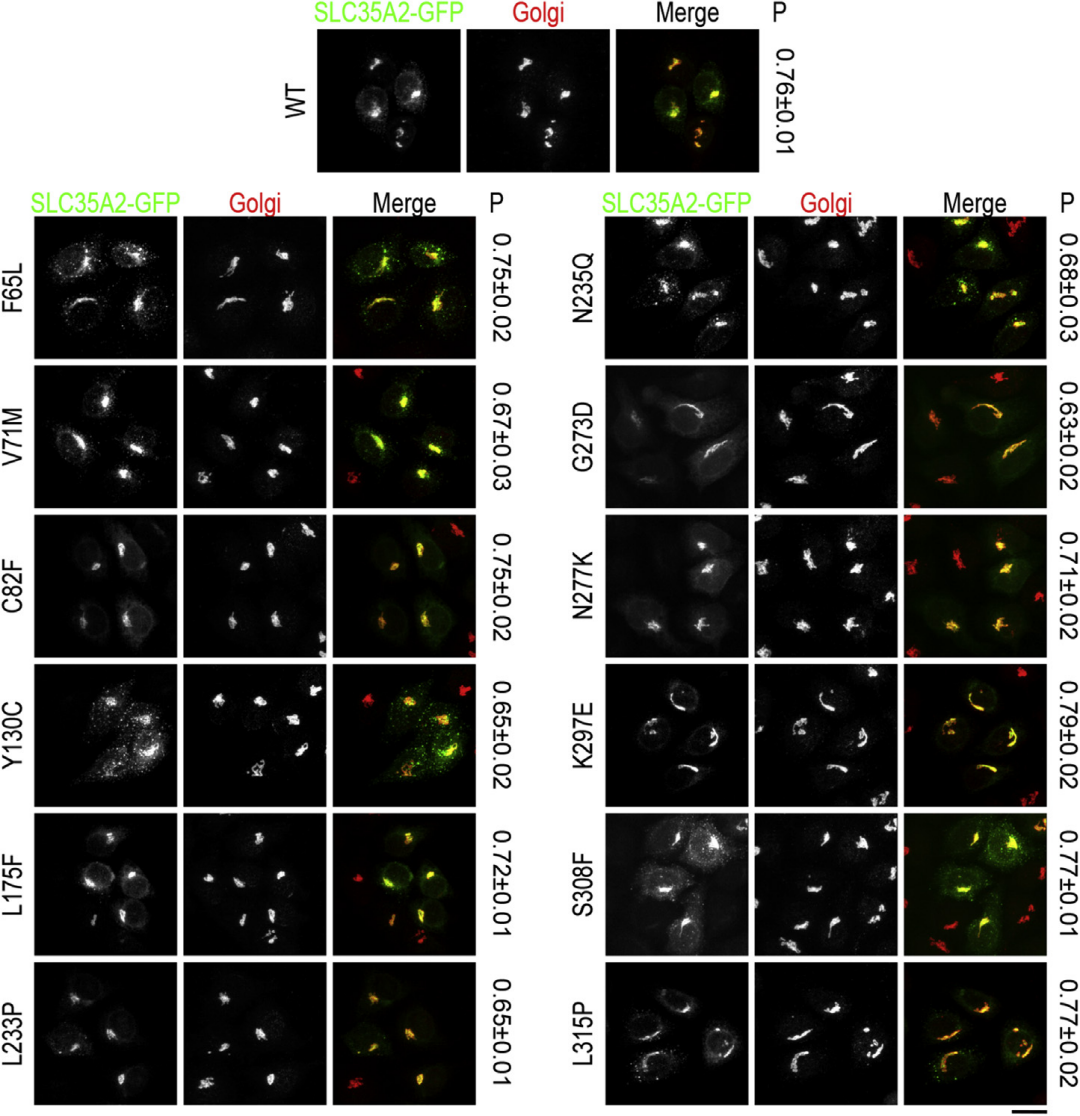
**Localization of disease-associated SLC35A2 mutants in *ΔSLC35A2* cells.***ΔSLC35A2* cells were transfected with SLC35A2-GFP constructs as indicated and further processed as described in Figure 2. “P” denotes values for the Pearson's coefficient for colocalization between two channels (mean ± SEM, n ≥ 28 cells per SLC35A2 construct). Scale bar, 20 μm.

**Figure 7:**
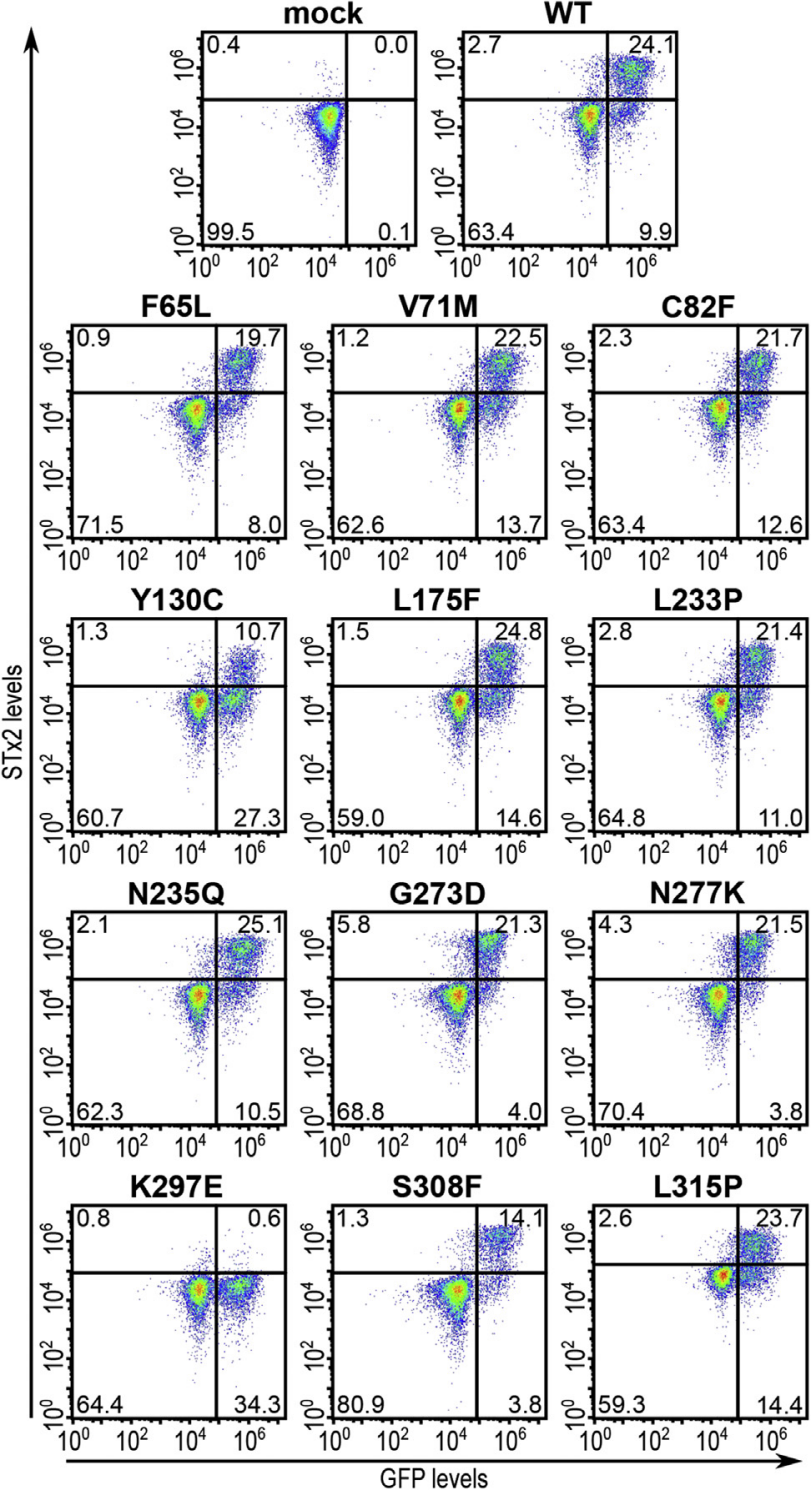
**Functional analyses of disease-associated SLC35A2 mutants.***ΔSLC35A2* cells were mock-transfected or transfected with indicated SLC35A2-GFP constructs and processed for STx2B binding after 24 h. Data from one replicate is depicted here and from multiple replicates are quantified in Figure 4.

Next, we analyzed 12 other point mutations of SLC35A2 that are associated with human diseases (Fig. 5, *A*–*E*) (25, 26, 27, 28). Except for F65, all other disease-associated residues reside in the transmembrane helices of SLC35A2, and five (V71, C82, Y130, N235, and K297) are in close vicinity to the central cavity (Fig. 5, *A*–*E*). Pearson's colocalization revealed that all mutants localized to the Golgi apparatus (Fig. 6). In rescue experiments, seven mutants, V71M, C82F, Y130C, L175F, L233P, N235Q, and L315P, had reduced activities while the K297E mutation abolished activity altogether (Fig.7; quantification in Fig. 4). Thus, residue K297 is obligatorily required for SLC35A2 activity, and residues V71, C82, Y130, L175, L233, N235, and L315 are required for optimal activity. The requirement of K297 identified here is consistent with our previous observations showing that the K297A mutation substantially reduced SLC35A2 activity (10). Similarly, the inability of N235Q to rescue is consistent with the reduced activity of the N235A mutant observed in Figures 3 and 4.

### A three-pocket model may explain mechanisms of substrate interaction/specificity of SLC35A2 and SLC35A1

**Figure 8:**
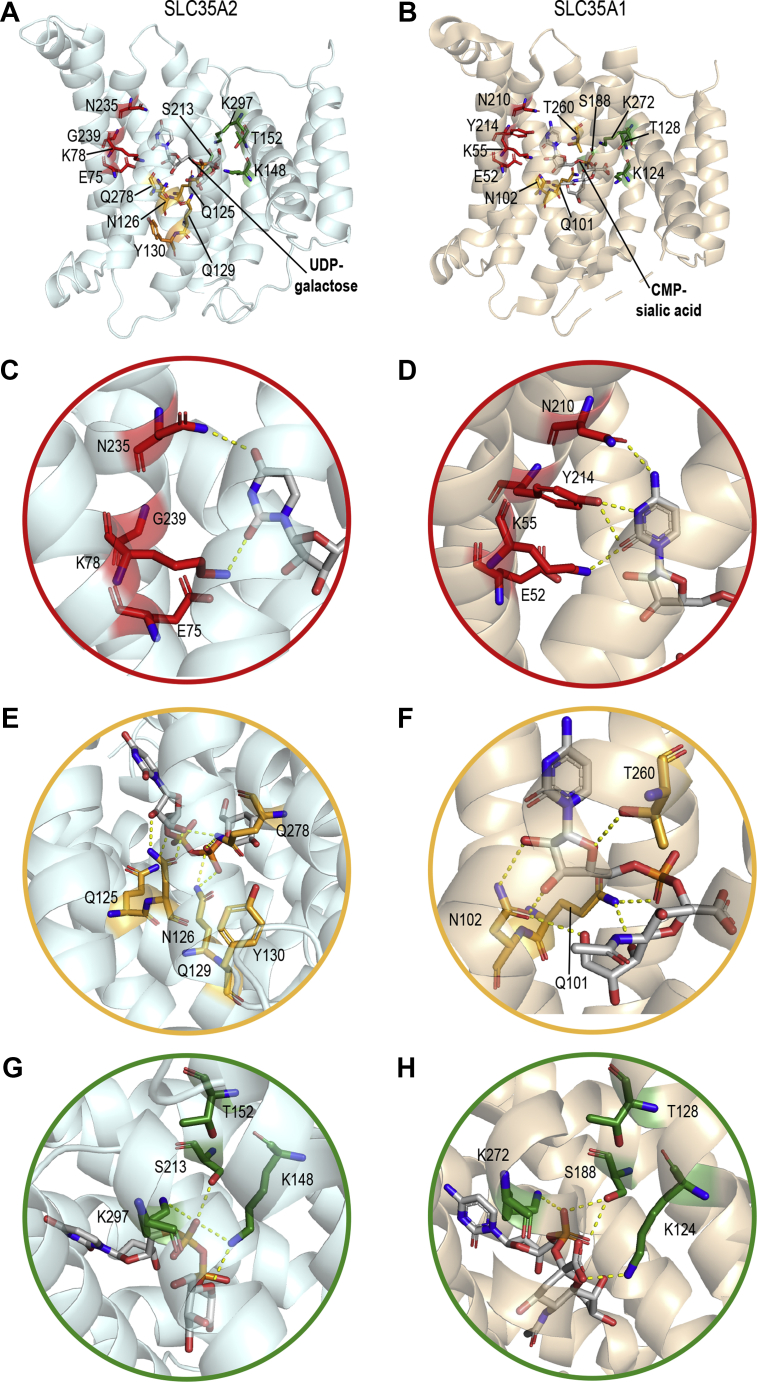
**Three-pocket model for SLC35A2 and SLC35A1.***A*, Cartoon depiction of the predicted structure of SLC35A2 with computationally docked substrate UDP-galactose. Residues composing the nucleobase, middle, and sugar pockets are shown as *sticks* and colored *red*, *orange*, and *green*, respectively. UDP-galactose is shown as *gray sticks*. *B*, Cartoon depiction of the crystal structure of SLC35A1 complexed with its substrate CMP-sialic acid (PDB: 6OH3). Color coding is similar to *A*. Note that residue N210 of SLC35A1 is depicted because it corresponds to the SLC35A2 required residue N235 and expected to interact with the substrate, but is not required for activity in our assays. *C–H*, zoom-in of SLC35A2 or SLC35A1 pockets. *Yellow dashed lines* indicate possible polar interactions.

We then aimed to gain a more general understanding of the structural requirements for SLC35A2 activity by combining results obtained here with those from our previous study (10). Residues that have robust effects on SLC35A2 activity (Y130, K148, and K297 from this study (Figs. 3, 4, and 7), and K78, S213, and K297 from our ref. 10) appear to reside in three physically separated regions (Fig. 8*A*, note this figure shows the docked substrate and more required residues identified later; see below). Notably, in a resolved structure of murine SLC35A1 complexed with its substrate CMP-sialic acid, the CMP-sialic acid adopts an extended conformation, with its cytosine binding the SLC35A2 K78 counterpart (K55 in SLC35A1), ribose and phosphate group in the middle, and sialic acid binding the K148 counterpart (K124 in SLC35A1) (23) (Fig. 8, *B*, *D*, and *H*, note here that Figure 8*B* shows required residues of SLC35A1 identified by us later, see below). We computationally docked UDP-galactose into the predicted structure of SLC35A2 and observed that the predicted orientation of UDP-galactose in SLC35A2 was similar to that of CMP-sialic acid in SLC35A1 (Fig. 8, *A* and *B*). The thematic similarities in substrate orientation led us to hypothesize that there are three pockets within the transporters responsible for interaction with nucleobases, ribose and/or phosphate groups, and sugars, respectively (Fig. 8, *A* and *B*). We use the terms “nucleobase pocket,” “middle pocket,” and “sugar pocket” to refer to those three pockets hereafter.

### Validation of the three-pocket model for SLC35A2

**Figure 9:**
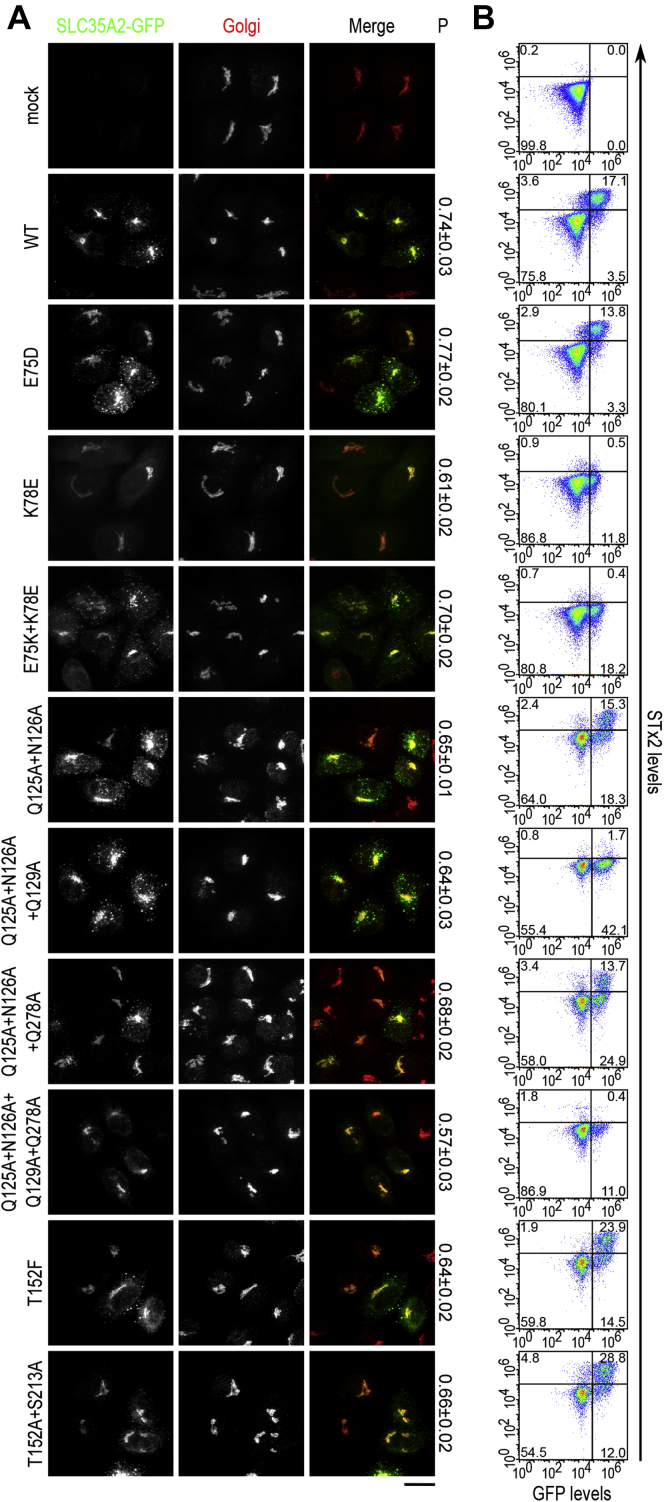
**Functional analyses of SLC35A2 mutants within the three pockets.***A*, immunofluorescence assay to detect overlap of indicated SLC35A2 constructs with the Golgi apparatus in *ΔSLC35A2* cells as described in Figures 2 and 6. “P”, Pearson's coefficient (mean ± SEM, n = 28 cells per SLC35A2 construct). Scale bar, 20 μm. *B*, *ΔSLC35A2* cells were mock-transfected or transfected with indicated SLC35A2-GFP constructs and processed for STx2B binding after 24 h. Data from one replicate is depicted here and from multiple replicates are quantified in Figure 4.

To test the above hypothesis, we first assayed for the requirement of residues predicted to contribute to the above three pockets in SLC35A2. The nucleobase pocket of SLC35A2 is formed by residues E75, K78, N235, and G239 (Fig. 8*C* and Table 1). Data presented in Figures 3, 4, and 7 (for E75, N235, and G239) and from our previous study (10) (for K78) already identified these residues as required for SLC35A2 function. We did not identify additional residues in proximity that may contribute to this pocket. Interestingly, E75 and K78 are one helical turn away from each other on the second transmembrane helix and could potentially form a salt bridge (Fig. 8*C*). To investigate the effect of charge and a potential role of an electrostatic interaction between the two residues, we maintained the charge of E75 (E75D), reversed the charge of K78 (K78E), or swapped the charges of the two residues (E75K+K78E). All three mutants localized to the Golgi (Fig. 9*A*). Unlike E75A, E75D had comparable activity to WT (Fig. 9*B*; quantification in Fig. 4), indicating that the charge of the E75 residue may be critical for activity. K78E lacked activity (Fig. 9*B*; quantification in Fig. 4), similar to K78A (10), suggesting that the side chain of the K78 residue is required. The double mutant E75K+K78E did not rescue (Fig. 9*B*; quantification in Fig. 4), suggesting that an electrostatic interaction between E75 and K78 may not be sufficient. Overall, the side chains of E75 and K78 are required.

**Table 1:** Corresponding SLC35A1 and SLC35A2 residues that contribute to the nucleobase, middle, and sugar pockets

SLC35A1	SLC35A2	Location
Nucleobase pocket		
E52	E75	TM 2
K55	K78	TM 2
	N235	TM 7
Y214	G239	TM 7
Middle pocket		
Q101	Q125	TM 3
N102	N126	TM 3
	Q129	TM 3
	Y130	TM 3
	Q278	TM 8
T260		TM 8
Sugar pocket		
K124	K148	TM 4
T128	T152	TM 4
S188	S213	TM 6
K272	K297	TM 9

We did not analyze SLC35A1 residues A105, F106, and A253 that correspond to Q129, Y130, and Q278 in SLC35A2. SLC35A1 residue N210 corresponds to SLC35A2 residue N235 and is not required for activity. SLC35A2 residue V285 corresponds to SLC35A1 residue T260 and is not required for activity.

In the middle pocket around Y130, the side chains of Q125, N126, Q129, and Q278 are accommodated in a common space within the central cavity (Fig. 8*E*). Alanine substitutions at these four residues individually have no effect on the activity of SLC35A2 (Figs. 3 and 4), but the orientation of these residues suggested they may be combinatorially required. To test this, we generated double, triple, and quadruple substitutions. All four new mutants localized to the Golgi (Fig. 9*A*), and importantly, the double, triple, or quadruple mutants had reduced activities (Fig. 9*B*; quantification in Fig. 4). Thus, like Y130, Q125, N126, Q129, and Q278 also contribute to the middle pocket of SLC35A2 and are required for optimal activity of SLC35A2 (Table 1).

K148, S213, and K297 are in the sugar pocket of SLC35A2 (Fig. 8*G* and Table 1). As described earlier, in this study, we observed that the S213A mutation has no effect on SLC35A2 activity (Figs. 3 and 4). However, we previously reported that the S213F mutation strongly inhibited activity (10). These findings suggest that the size of S213 may be critical. T152 is very close to S213 with the side chain pointing toward S213 (Fig. 8*G*), and the T152A mutation had no effect on the activity of SLC35A2 (Figs. 3 and 4). We hypothesized that the small sizes of both T152 and S213 are required for the activity of SLC35A2, while the identities of the side chains are less critical. To test this, we made T152F and T152A+S213A. Both mutants localized to the Golgi (Fig. 9*A*). Unlike T152A, T152F had reduced activity (Fig. 9*B*; quantification in Fig. 4). In contrast, the double mutant T152A+S213A had similar activity as WT (Fig. 9*B*; quantification in Fig. 4). These results align with our hypothesis that the size of side chains at positions 152 and 213 are critical and further indicate that T152 also contributes to the sugar pocket, in addition to K148, S213, and K297 (Table 1).

### Confirmation of results using Gb3 expression and STx1B binding as independent assays

**Figure 10:**
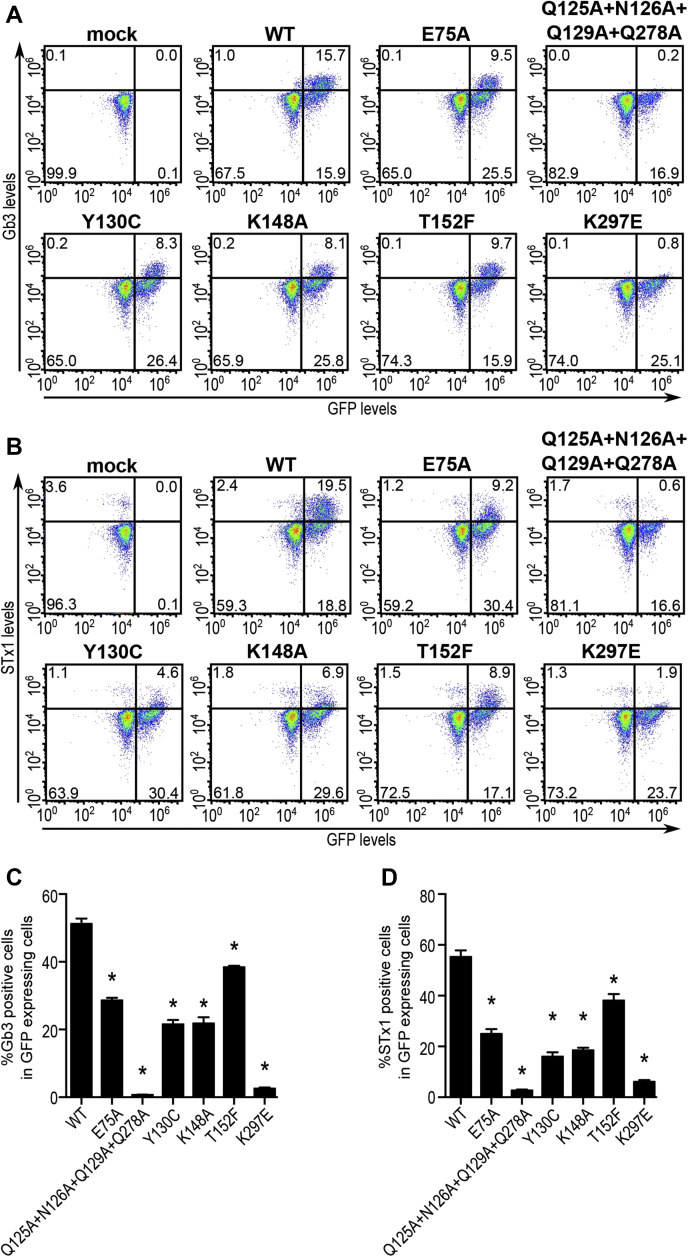
**Validation of SLC35A2 mutants using Gb3 expression and STx1B binding.***A* and *B*, *ΔSLC35A2* cells were mock-transfected or transfected with indicated SLC35A2-GFP constructs and analyzed for Gb3 expression (*A*) or STx1B binding (*B*) 24 h posttransfection. *C* and *D*, in GFP-expressing cells from *A* and *B*, cell percentage positive for Gb3 (*C*) or STx1B binding (*D*) were quantified (mean ± SEM; n = 3; ∗*p* < 0.05 for the comparison between WT SLC35A2-GFP and all other constructs by one-way ANOVA and Dunnett's *post hoc* test).

To independently validate that the reduced STx2B binding is due to decreased activities of SLC35A2 mutants, we repeated the rescue experiment for selected constructs using Gb3 expression and STx1B binding as independent end points. Gb3 expression provides a more direct measure of SLC35A2 transport activity because it depends directly on UDP-galactose transport. Notably, for all mutants included in this confirmatory experiment, profiles of Gb3 expression and STx1B binding were similar to that of STx2B binding (Fig. 10, *A*–*D*). Thus, three independent assays confirmed the requirements of residues required for SLC35A2 activity identified in this study.

### Validation of the three-pocket model for SLC35A1 function

**Figure 11:**
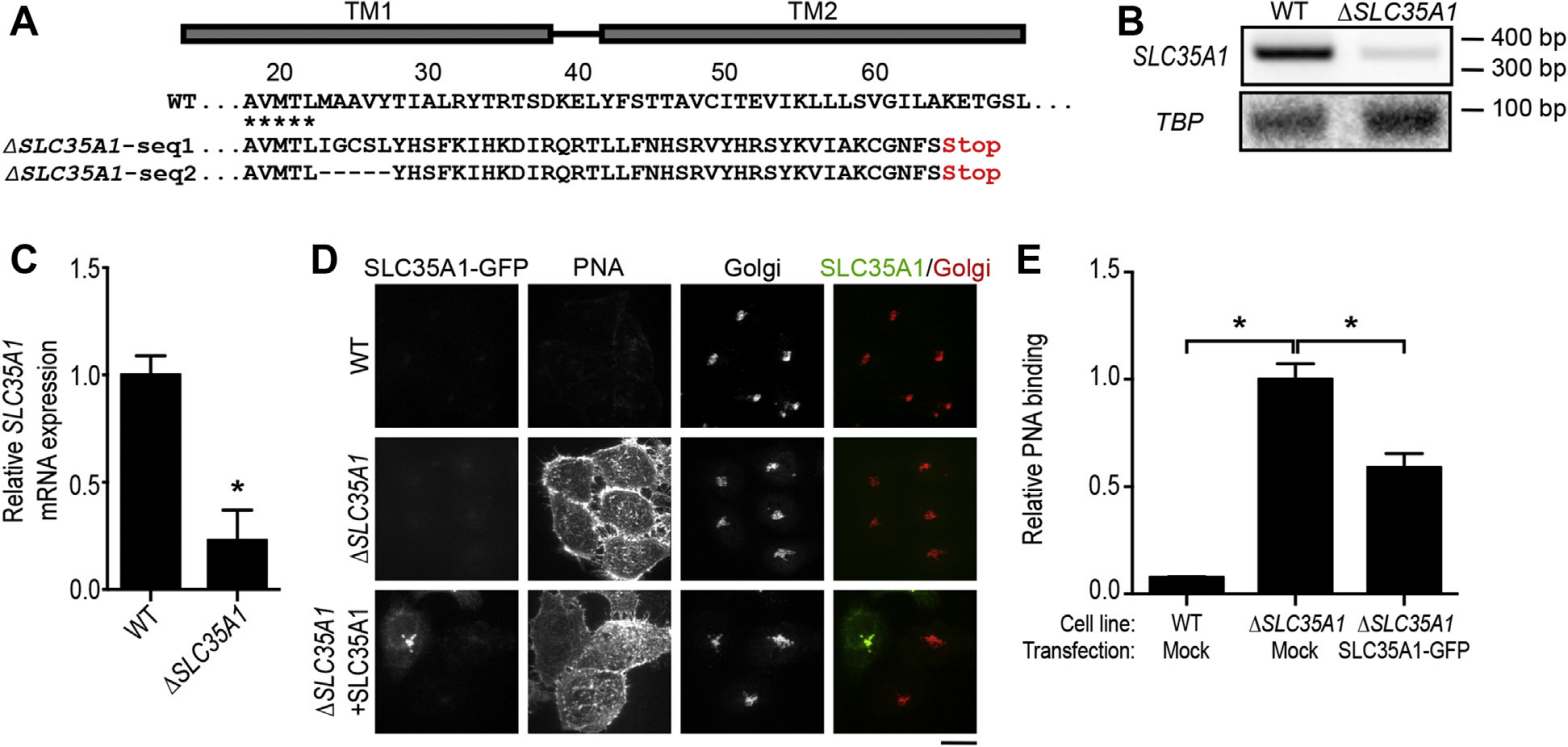
**Expression of SLC35A1-GFP rescues the phenotype of *ΔSLC35A1* cells.***A*, amino acid sequence of SLC35A1 in WT and *ΔSLC35A1* cells. Area with asterisks shows alignment between WT and *ΔSLC35A1* cells. *B*, RT-PCR. *C*, qRT-PCR to quantify the relative levels of *SLC35A1* mRNA in WT or *ΔSLC35A1* cells. Levels in WT cells were normalized to 1 (mean ± SE; n = 3; ∗*p* < 0.05 by *t* test). *D*, mock-transfected WT or *ΔSLC35A1* cells or SLC35A1-GFP transfected *ΔSLC35A1* cells were processed to detect GFP, PNA binding, and the Golgi apparatus 24 h posttransfection. Scale bar, 20 μm. *E*, intensity of bound PNA from *D*. Levels in mock-transfected *ΔSLC35A1* cells were normalized to 1 (mean ± SE; n = 28 cells per condition; ∗*p* < 0.05 for the comparison between mock-transfected *ΔSLC35A1* cells and other groups by one-way ANOVA and Dunnett's *post hoc* test). Seq., sequence; TM, transmembrane helix.

Finally, we sought to test whether the three-pocket model may also be applied to SLC35A1 (Fig. 8*B*). Deficiency in SLC35A1 leads to the lack of N-glycan sialylation, which exposes galactose on the cell surface as a recognition site for peanut agglutinin (PNA). Therefore, we used a well-characterized PNA binding assay to assess function of SLC35A1 mutants (14, 15, 29). For our experiments, we generated a clonal *ΔSLC35A1* line using a lentiviral-based CRISPR/Cas9 method. Premature stop codons introduced into the second transmembrane domain significantly reduced *SLC35A1* mRNA level when compared with WT cells (Fig. 11, *A*–*C*). *ΔSLC35A1* cells bound significantly higher levels of PNA than WT cells (Fig. 11, *D* and *E*). Transiently expressed SLC35A1-GFP construct localized to the Golgi and reduced PNA-binding in *ΔSLC35A1* cells (Fig. 11, *D* and *E*). The ability of SLC35A1-GFP to rescue was comparable to FLAG-tagged SLC35A1 (Fig. S1, *C* and *D*), implying that the GFP tag did not interfere with activity.

**Figure 12:**
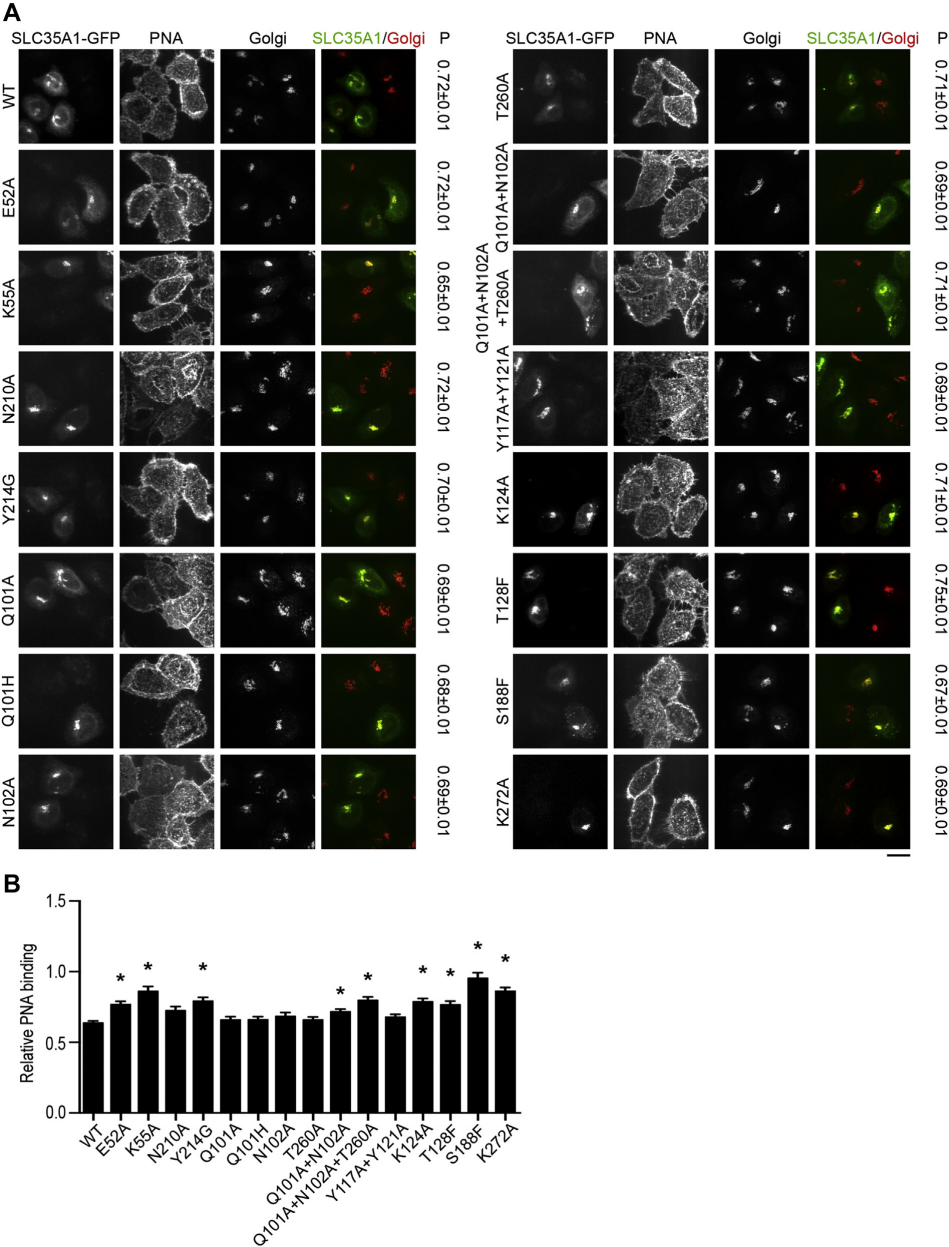
**Functional analyses of SLC35A1 mutants.***A* and *B*, *ΔSLC35A1* cells were mock-transfected or transfected with indicated SLC35A1-GFP constructs and processed to detect GFP, PNA binding, and the Golgi apparatus. “P” Pearson's coefficient for colocalization between GFP and the Golgi apparatus (mean ± SEM, n > 100 cells per SLC35A1 construct). Scale bar, 20 μm. Relative intensity of bound PNA is quantified in *B*. For each construct, levels in nontransfected *ΔSLC35A1* cells were independently normalized to 1 (mean ± SE; n > 100 cells per SLC35A1 construct; ∗*p* < 0.05 for the comparison between WT SLC35A1-GFP and all other constructs by one-way ANOVA and Dunnett's *post ho*c test).

We then generated a set of mutants to test whether SLC35A1 residues that contribute to the three pockets are required for activity (Table 1). This assay included SLC35A1 residues that directly corresponded to residues in SLC35A2, which formed the three pockets (E52, K55, N210, Y214, Q101, N102, K124, T128, S188, and K272); SLC35A1 residues predicted to interact with the substrate based on the crystal structure, but for which corresponding SLC35A2 counterparts were not required for activity (T260, Y117, and Y121 in SLC35A1, corresponding to V285, F141, and Y145 in SLC35A2); and a disease-causing SLC35A1 mutant Q101H (30). All mutants expressed in *ΔSLC35A1* cells correctly localized to the Golgi (Fig. 12*A*). Nucleobase pocket mutant N210A, middle pocket mutants Q101A, Q101H, N102A, and T260A, and sugar pocket mutants Y117A+Y121A reduced PNA-binding levels, similar to WT (Fig. 12, *A* and *B*). In contrast, nucleobase pocket mutants E52A, K55A, and Y214G, middle pocket mutants Q101A+N102A and Q101A+N102A+T260A, and sugar pocket mutants K124A, T128F, S188F, and K272A did not reduce PNA binding (Fig. 12, *A* and *B*). Thus, E52, K55, Y214, K124, T128, S188, and K272 are critical for SLC35A1 activity (Table 1). Though single mutations at site Q101, N102, or T260 did not affect SLC35A1 activity, the fact that Q101A+N102A and Q101A+N102A+T260A had reduced activities suggests that the combined activities of these residues are required (Table 1). Overall, our results imply that the three-pocket model is applicable to define residues required for activity of SLC35A1 and SLC35A2.

## Discussion

We proposed a three-pocket model for substrate coordination by nucleotide sugar transporters SLC35A1 and SLC35A2, and utilized this model to identify potential elements that determine the substrate specificity of the two transporters.

In the nucleobase pockets, both E52 (for SLC35A1)/E75 (for SLC35A2) and K55 (for SLC35A1)/K78 (for SLC35A2) are required. K55 (for SLC35A1)/K78 (for SLC35A2) may coordinate with cytosine/uracil while E52 (for SLC35A1)/E75 (for SLC35A2) may play a role in stabilizing the orientation of K55 (for SLC35A1)/K78 (for SLC35A2) (Fig. 8, *C* and *D*). The Y214G mutation in SLC35A1 and G239Y mutation in SLC35A2 reduced activity (Figs. 3, 4, and 12, *A* and *B*). These residues reside on the seventh transmembrane domain, which has been reported to be particularly important for recognition of different nucleotides (20, 21, 31). In SLC35A1-SLC35A2 chimeric transporters, the seventh transmembrane helix derived from SLC35A1 is sufficient to confer CMP-sialic acid specificity in an otherwise SLC35A2 context (14, 15, 32). Ahuja and Whorton previously hypothesized that Y214 of SLC35A1 is the structural basis for not recognizing uracil because Y214 may not form a strong hydrogen bond with the protonated N-3 amine of uracil (23). Our results further support this idea by showing that tyrosine at position 239 of SLC35A2 is not tolerated. Another interesting finding in the nucleobase pocket was the difference in the requirement for N210 in SLC35A1 and the corresponding N235 in SLC35A2. Although the side chain of N210 (for SLC35A1) is expected to interact with cytosine (Fig. 8*D*), this interaction is likely not required for function because N210A did not affect SLC35A1 activity (Fig. 12, *A* and *B*). But, mutation of N235 in SLC35A2 to either alanine or glutamine slightly reduced SLC35A2 activity (Figs. 3, 4, and 7), implying that N235 in SLC35A2 is necessary (Fig. 8*C*). The differential requirements of N210 for SLC35A1/N235 for SLC35A2 may relate to the fact that Y214 for SLC35A1 likely interacts with cytosine, but the corresponding G239 for SLC35A2 is not expected to interact with uracil. For SLC35A2, the lack of interaction between G239 and uracil may necessitate an interaction between N235 and uracil, making N235 required for activity.

In the middle pockets, Q101 (for SLC35A1)/Q125 (for SLC35A2) may coordinate with the α-phosphate and N102 (for SLC35A1)/N126 (for SLC35A2) may coordinate with the ribose (Fig. 8, *E* and *F*). In contrast to SLC35A1, whose substrate is monophosphate, SLC35A2 has an extra required pair of glutamine residues (Q129 and Q278), which may be required to coordinate with the β-phosphate of UDP-galactose (Fig. 8*E*). The corresponding residues in SLC35A1 are both alanine (A105 and A253), which do not directly interact with CMP-sialic acid. Thus, A105 (for SLC35A1)/Q129 (for SLC35A2) and A253 (for SLC35A1)/Q278 (for SLC35A2) may be the structural determinant for substrate specificity in the middle pocket. The importance of these two glutamine residues has been underappreciated, probably because most of the previous studies focused on CMP-sialic acid transporters, the substrate of which is a monophosphate.

In the sugar pocket, both K272 (for SLC35A1)/K297 (for SLC35A2) and S188 (for SLC35A1)/S213 (for SLC35A2) are expected to coordinate with the α-phosphate (Fig. 8, *G* and *H*). The relatively small side chains of S188 (for SLC35A1)/S213 (for SLC35A2) and T128 (for SLC35A1)/T152 (for SLC35A2) may be designed to allow this accommodation (Fig. 8, *G* and *H*). The observations that fewer sugar-coordinating residues are required than those coordinating the nucleobase, ribose, and phosphate groups align with previous findings that substrate recognition by nucleotide sugar transporters mostly depends on the recognition of the nucleotide rather than the sugar (4, 20, 21, 22, 23, 31).

The disease-associated SLC35A2 mutation K297E abolished activity, providing a straightforward mechanism of disease. Several other disease-associated SLC35A2 mutations (V71M, C82F, Y130C, L175F, L233P, N235Q, and L315P) also inhibited SLC35A2 activity, and the extent of inhibition might be sufficient to induce disease. However, four disease-associated SLC35A2 mutants (F65L, G273D, N277K, and S308F) and one disease-associated SLC35A1 mutant (Q101H) did not show any decrease in activities. The means by which these mutations induce disease cannot be explained by simple loss-of-function of transporter activity. A possible explanation could be that small changes in glycosylation induce as yet unclear downstream consequences that are responsible for disease development.

Findings described here are potentially of broad relevance. The insights we obtained about specificity determinants in SLC35A1 and SLC35A2 improve understanding of the structure–function relationship of this important family of transporters. In particular, the three-pocket model may aid the prediction of the pathogenicity of certain SLC35A1 or SLC35A2 variants that have mutations within or near the pockets. For example, disease-associated SLC35A2 mutations K78R (33) and ΔL296 (34) are probably pathogenic because K78 is within the nucleotide pocket and L296 is close to the sugar pocket of SLC35A2. Similarly, it should be possible to expand this model to understand how other NSTs achieve substrate specificity. Indeed, our preliminary analysis of the predicted structure of SLC35A3 suggests that the three pockets are conserved. Furthermore, our work may be leveraged to develop specific inhibitors of SLC35 family proteins. As previously described, SLC35A2 is a required host factor for STx2 toxicosis and depletion of SLC35A2 blocks STx2B surface binding and protects against STx2 toxicity (9, 10). Notably, there is a high interest in developing small-molecule inhibitors of STx2 because antibiotic therapy is contraindicated for treatment of infections caused by STx2-producing *Escherichia coli* and antidotes are not available (35, 36, 37, 38, 39, 40, 41). Structure–activity relationship studies on two classes of compounds that block STx2 trafficking, tamoxifen compounds, and retro-2 molecules, have been reported (38, 39, 41, 42, 43). Conceivably, our studies may lead to the development of a specific SLC35A2 inhibitor that transiently, but effectively, blocks SLC35A2 function in renal cells, which represent the target of STx2 *in vivo* (44). Another possibility of targeting NSTs for therapy is blocking SLC35A1 for treating cancer. Cell surface hypersialylation benefits tumor cell growth and metastasis (12), making SLC35A1 an attractive target for drug design. Indeed, a small-molecule inhibitor of SLC35A1 has been shown to reduce cell surface sialylation and inhibit the metastasis of human colorectal cancer cells within a mouse model (45). Our findings may aid the development of new and specific SLC35A1 inhibitors for chemotherapeutic use.

To summarize, our results suggest that three separate pockets may coordinate with different substrate moieties in SLC35A1 and SLC35A2, providing a modular mechanism for substrate recognition. Since SLC35A1 and SLC35A2 are most distantly related in the known family of mammalian NSTs (46), it seems likely that the structural motifs identified here are of general importance for substrate recognition throughout the NST family.

## Experimental procedures

### Plasmids and DNA constructs

We described the SLC35A2-GFP plasmid previously (10). A template plasmid from DNASU (clone HsCD00357880) was used to generate the SLC35A1-GFP construct. Clone HsCD00357880 codes for the best-characterized isoform of SLC35A1, isoform a, which is referred to as WT SLC35A1. We moved the WT SLC35A1 insert into pEGFP-N3 vector using 5′-GAGCTCAAGCTTATGGCTGCCCCGAGAGAC-3′ and 5′-GGCGATGGATCCCACACCAATAACTCTCTCCTTTGAAGCTG-3′ primers and digesting the product with *Hind*III and *BamH*I (NEB) enzymes. Point mutations were introduced into the above plasmids using QuickChange. FLAG-tagged SLC35A2 and SLC35A1 were constructed by moving SLC35A2 and SLC35A1 inserts into the *Hind*III and *BamH*I sites of a pCMV-FLAG-SLC30A10 plasmid that we previously described (47). All plasmids were verified by sequencing.

### Cell culture and DNA transfections

All experiments were performed using HeLa cells. WT cells refer to a HeLa subline that overexpresses Gb3 synthase, which induces overexpression of Gb3 on cell surface and has been described extensively previously (9, 10, 48). Cell culture and DNA transfections were performed exactly as described before (9, 10, 48).

### Generation of knockout cell clones

*ΔSLC35A2* cells were described previously (10). *ΔSLC35A1* cells were generated using a third-generation lentivirus-based CRISPR/Cas9 system extensively described by us previously (9, 10). The guide RNA sequence for SLC35A1 was 5′-GGUAUAGACUGCAGCCAUCA-3′ and targets the second exon of the *SLC35A1* gene, which codes for amino acids 22–28 (full-length SLC35A1 isoform a has 337 amino acids). After infection, single cell clones were selected, propagated, and genomic DNA extracted and sequenced to confirm that stop codons were introduced into *SLC35A1* (9, 10).

### RT-PCR and qRT-PCR

These were performed as described by us previously (9, 10, 48). The primer pair used to amplify *SLC35A1* was 5′-CAACCACAGCCGTGTGTATCA-3′ (forward) and 5′-AAGCGTAACTCCAGCACACA-3′ (reverse). Primers to amplify *TBP* were 5′-CGAACCACGGCACTGATTTTC-3′ (forward) and 5′-TTTCTTGCTGCCAGTCTGGAC-3′ (reverse). Transcript levels were quantified using the ΔΔC_T_ method (49).

### Fluorescence-activated cell sorting (FACS) analyses

FACS analyses were performed exactly as previously described (10). Cell populations were manually gated based on their distribution.

### Structure prediction and presentation

Predicted structure of SLC35A2 was generated using the PHYRE 2.0 server (24) based on a recently solved crystal structure of murine SLC35A1 (PDB 6OH3) (23). All protein structure images were generated using the open-source software PyMOL (Schrödinger, LLC, 2018). Computational substrate docking was done using the UCSF Chimera program (https://www.cgl.ucsf.edu/chimera/) and the AutoDock Vina tool (50).

### Immunofluorescence microscopy

For immunofluorescence without binding of STx2B or PNA, cells grown on cover slips were fixed with 3.7% formaldehyde and processed for immunofluorescence using anti-giantin and/or anti-FLAG primary antibodies and appropriate secondary antibodies essentially as described previously (10, 51).

Immunofluorescence to detect STx2B binding was performed exactly as previously described (10). For PNA-binding assays, cells were incubated with 5 μg/ml Alexa Fluor 568 conjugated PNA lectin (ThermoFisher) in phosphate-buffered saline containing 1% bovine serum albumin on ice for 30 min. After this, cells were washed in ice-cold phosphate-buffered saline, fixed with 3.7% formaldehyde, and processed as described above.

All images were captured using a Nikon swept-field confocal microscope equipped with a four-line high-power laser launch and a 100 × 1.45 NA oil immersion objective. The camera was an iXon3 X3 DU897 electron-multiplying charge-coupled device camera (Andor Technology). All images were captured as z-stacks with 0.2-μm spacing between individual frames.

### Image analyses

Image quantification was performed using NIH ImageJ. For each experiment, all images were captured using the same settings. All depicted images in the figures are maximum-intensity projections of the stacks.

To quantitatively assess the colocalization of each SLC35A2 or SLC35A1 mutant with a Golgi marker, we calculated Pearson's colocalization coefficient using all Z-stacks. For each cell, the outline was drawn to cover the largest expanse of the cell being analyzed, and the Pearson's coefficient was calculated between two signals. Constructs with a Pearson's co-efficient > 0.5 were considered Golgi-localized (10).

To quantify the relative intensity of PNA signal per cell, an average projection was first generated. The background was subtracted, the cell was outlined, and total fluorescence intensity measured.

### Statistical analyses

Prism 8 software (GraphPad Software) was used for all statistical analyses. All experiments were repeated at least three times independently. Comparisons between two groups were performed using *t* test. Comparisons between multiple groups were performed using one-way ANOVA followed by Dunnett's *post hoc* test. For all analyses, *p* < 0.05 was considered to be statistically significant. Asterisks in graphs, where present, denote statistically significant differences.

## Data availability

All data presented in this paper are contained within this manuscript.

## Supporting information

This article contains supporting information.

## Conflict of interest

The authors declare that they have no conflicts of interest with the contents of this study.
