# Supplementary material for: A three-pocket model for substrate coordination and selectivity by the nucleotide sugar transporters SLC35A1 and SLC35A2
Source: J Biol Chem. 2021 Aug 10;297(3):101069. doi: 10.1016/j.jbc.2021.101069 (PMC8411240; doi:10.1016/j.jbc.2021.101069)
Supplement: Figure S1 [file mmc1.pdf]

SUPPORTING INFORMATION FOR

A three-pocket model for substrate coordination and selectivity by the nucleotide sugar transporters SLC35A1 and SLC35A2

Danyang Li[1] and Somshuvra Mukhopadhyay[1*]

[1] Division of Pharmacology & Toxicology, College of Pharmacy, Institute for Cellular & Molecular Biology, and Institute for Neuroscience, The University of Texas at Austin, Austin, TX 78712

* To whom correspondence should be addressed: Somshuvra Mukhopadhyay, Associate Professor, Division of Pharmacology & Toxicology, The University of Texas at Austin, 3.510E BME, 107 W. Dean Keeton, Austin, TX 78712. E-mail: som@austin.utexas.edu

This PDF contains
Supplemental Fig.S1

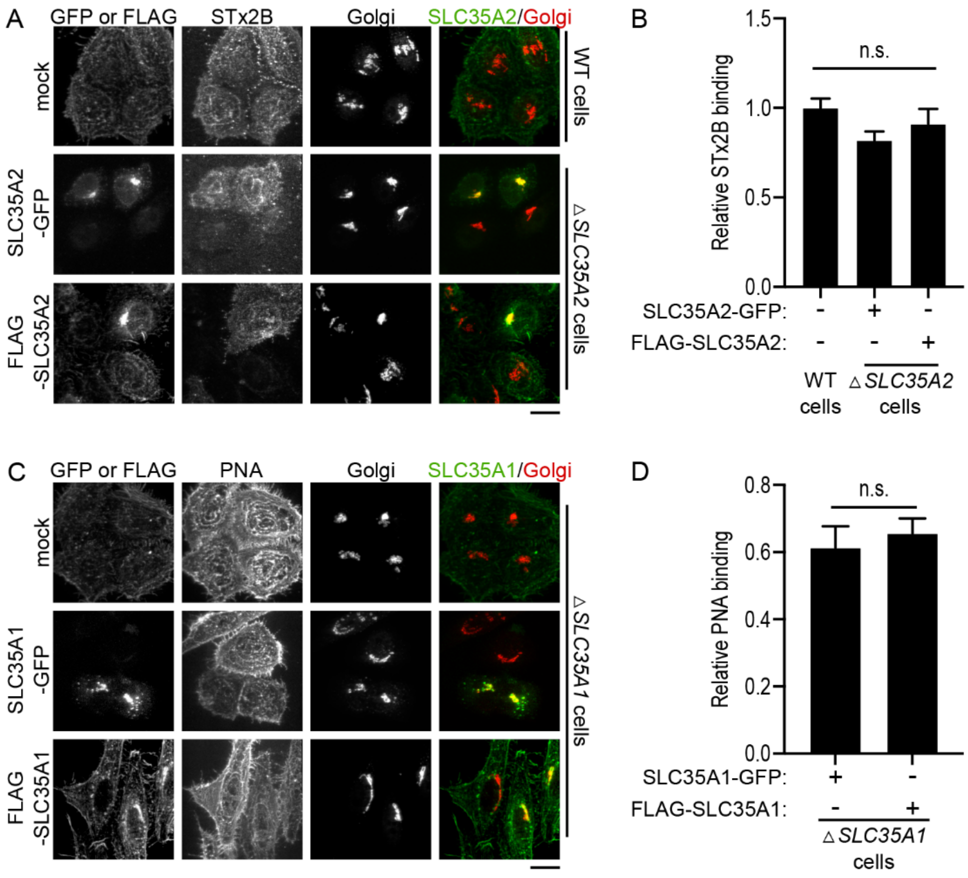

Figure S1. Comparison of rescue by FLAG- or GFP-tagged SLC35A2 and SLC35A1.

A. STx2 binding was performed in mock-transfected WT cells or *ΔSLC35A2* cells transfected with indicated SLC35A2 constructs as described in the *Methods*. Cultures were then processed to detect GFP or FLAG, STx2B, and the Golgi apparatus. Signal in mock-transfected cells for GFP/FLAG is background signal of the FLAG antibody, which was included in the staining. Scale bar, 20 µm.

B. Quantification of relative STx2B levels from ***A***. Levels in WT cells were normalized to 1. Mean ± SE. N = 28 cells per condition. N.S., not significant by one-way ANOVA.

C. *ΔSLC35A1* cells were mock transfected or transfected with indicated SLC35A1 constructs and processed to detect GFP or FLAG, PNA binding, and the Golgi apparatus. Signal in mock-transfected cells for GFP/FLAG is background signal of the FLAG antibody included in the staining. Scale bar, 20 µm.

D. Quantification of relative intensity of bound PNA from ***C***. For each construct, levels in non-transfected *ΔSLC35A1* cells were independently normalized to 1. Mean ± SE. N = 28 cells per construct. N.S., not significant by *t*-test.
